# Comparison of gasless transaxillary endoscopic thyroidectomy, endoscopic thyroidectomy via areola approach and conventional open thyroidectomy in patients with unilateral papillary thyroid carcinoma

**DOI:** 10.1186/s12957-024-03433-2

**Published:** 2024-06-05

**Authors:** Yu Ding, Chenjie Qiu, Chunfu Zhu, Yuan Li, Xiang Geng, Guojun Lv, Xiaoyi Yan, Feng Ju, Shijia Wang, Wenze Wu

**Affiliations:** 1 Department of Thyroid Surgery, The Affiliated Changzhou No.2 People’s Hospital of Nanjing Medical University, Changzhou Jiangsu, 213000 China; 2 Department of General Surgery, Changzhou Hospital of Traditional Chinese Medicine, Changzhou Jiangsu, 213000 China

**Keywords:** Papillary thyroid carcinoma, Transaxillary approach, Areola approach, CUSUM

## Abstract

**Background:**

Gasless transaxillary endoscopic thyroidectomy (GTET) and endoscopic thyroidectomy via the areola approach (ETA) have emerged as minimally invasive surgical techniques for managing papillary thyroid carcinoma (PTC). This study aimed to assess the surgical efficacy of endoscopic thyroidectomy (ET) as compared to conventional open thyroidectomy (COT) in PTC patients.

**Methods:**

Between 2020 and 2022, 571 PTC patients underwent unilateral thyroidectomy accompanied by ipsilateral central lymph node dissection. This cohort comprised 72 patients who underwent GTET, 105 ETA, and 394 COT. The analysis encompassed a comprehensive examination of patient clinicopathologic characteristics and postoperative complaints. Furthermore, the learning curve of GTET was evaluated using the cumulative summation (CUSUM) method.

**Results:**

Patients in the ET group exhibited a lower mean age and a higher proportion of female individuals. Operation time in the ET group was significantly longer. No significant differences were observed in the incidence of postoperative complications among the three groups. With regard to postoperative complaints reported three months after surgery, GTET demonstrated superior alleviation of anterior chest discomfort and swallowing difficulties. Patients who underwent ET reported significantly higher cosmetic satisfaction levels. Additionally, the learning curve of GTET was 27 cases, and the operation time during the mature phase of the learning curve exhibited a significant reduction when compared to ETA.

**Conclusions:**

The findings of this study affirm the safety and feasibility of employing GTET and ETA for the surgical management of PTC. GTET presents an attractive surgical option, particularly for patients with unilateral PTC who place a premium on cosmetic outcomes.

## Introduction

Papillary thyroid carcinoma(PTC) is the most common malignancy in the realm of endocrine malignancies and surgical intervention remains the foremost therapeutic approach for managing PTC [1, 2]. Conventional open thyroidectomy(COT) offered the advantage of being intuitive and convenient but came with the significant drawback of leaving an evident surgical scar on the anterior neck, often colloquially referred to as the "suicide incision." This conspicuous scar could negatively impact patients' quality of life. Over the past few decades, surgeons have been dedicated to exploring alternatives that would minimize scarring and reduce the length of the surgical incision in thyroid surgery.

In 1997, a significant milestone was achieved when Huscher conducted the pioneering procedure of endoscopic thyroidectomy(ET) [3]. Initially, ET was primarily considered suitable for the removal of benign thyroid tumors. However, as experience accumulated and the favorable prognosis of PTC became apparent, its application expanded to include thyroid malignancies [4, 5].

Among the various approaches to ET, the breast approach has emerged as one of the most widely adopted techniques [6–9]. Additionally, transaxillary thyroidectomy has gradually become popular in our country in recent years [10]. Nevertheless, the safety and feasibility of transareola thyroid surgery and transaxillary thyroid surgery for the treatment of thyroid carcinoma warrant further investigation. In our institution, COT and endoscopic thyroidectomy via the areola approach (ETA) have been routine procedures for several years. However, since September 2020, we have introduced a novel thyroidectomy technique known as gasless transaxillary endoscopic thyroidectomy (GTET). Although all three of these approaches have gained substantial popularity, they have seldom been simultaneously compared. Given our recent introduction of GTET and our journey toward proficiency, we applied the Cumulative Sum (CUSUM) method to construct a surgeon's learning curve for this technique. This analysis aimed to compare perioperative outcomes across various learning phases.

The primary objective of this study was to conduct a comparative analysis of the surgical outcomes associated with these three thyroidectomy approaches and to further assess the feasibility of GTET and ETA in the context of treating PTC.

## Materials and methods

### Study subjects

A retrospective analysis was conducted on clinical data obtained from 571 patients diagnosed with unilateral PTC who were admitted to Changzhou Second People's Hospital between September 2020 and December 2022. The lead surgeon possessed considerable expertise in COT and had accumulated over eight years of experience, performing more than 500 ETA surgeries. Inclusion criteria for patient enrollment were as follows: (1) age ranging from 18 to 55 years; (2) clinical diagnosis of unilateral malignant thyroid tumor with a tumor diameter of ≤ 2 cm; (3) absence of preoperative ultrasonographic evidence indicating lateral lymph node metastasis or local invasion; (4) no contraindications for surgery, such as cardiopulmonary insufficiency; and (5) no prior history of neck surgery or radiation therapy. Exclusion criteria for enrollment were as follows: (1) presence of bilateral lesions; (2) patients with hyperthyroidism; (3) tumor diameter > 2 cm; and (4) incomplete clinicopathological data.

Retrospectively, we meticulously collected patients' medical records and conducted an assessment of surgical outcomes, encompassing factors such as operation time, maximum tumor size, tumor multiplicity, presence of Hashimoto's thyroiditis, postoperative hospital stay, mean duration for removal of the surgical drain, the count of central lymph nodes retrieved, and the number of metastatic central lymph nodes. Surgical drains were removed if the daily drainage output was below 30 ml. Surgical complications, including temporary hoarseness, permanent hoarseness, parathyroid damage, wound infections, postoperative hematomas, and recurrences, were diligently assessed and compared across surgical groups. Parathyroid damage was quantified by measuring the difference in parathyroid hormone levels before and on the first day after surgery(ΔPTH). To monitor potential recurrences, neck ultrasonography was conducted at six-month intervals postoperatively.

Patients' postoperative pain in the neck and anterior chest, as well as swallowing discomfort, were assessed through a combination of a straightforward questionnaire and physician interviews. Additionally, postoperative cosmetic satisfaction was evaluated on a five-point scale, ranging from 1 (very satisfied) to 5 (very dissatisfied), with patients providing their ratings during outpatient clinic visits three months following the surgical procedure.

### Surgical procedures

#### COT

The patient was positioned in a supine orientation with their neck extended. A collar incision, measuring 5 cm in length, was precisely crafted approximately 2 cm above the sternal notch. The incision was made through the skin, and a subcutaneous skin flap was delicately dissected on the deep surface of the platysma muscle. This dissection extended upward to the thyroid cartilage, downward to the superior suprasternal fossa, and laterally to the medial one-third of the anterior border of the sternocleidomastoid muscle (SCM). Subsequently, a longitudinal incision was made at the midline of the neck through the anterior cervical fascia. The strap muscle was gently retracted, facilitating exposure of the thyroid gland. This exposure allowed for the visualization of the superior pole of the thyroid. Notably, the superior laryngeal nerve and superior parathyroid gland were carefully identified and preserved during this phase of the procedure. To further the dissection, the recurrent laryngeal nerve (RLN) was meticulously dissected and safeguarded following the division of the middle thyroid vein. Close attention was paid to preserving the inferior parathyroid gland located in proximity to the inferior pole of the thyroid. Once the thyroid gland was adequately mobilized and separated from the trachea, it was excised and sent for frozen section analysis. In cases where the specimens were confirmed to be PTC, the ipsilateral central lymph nodes were systematically removed. For drainage purposes, a tube was threaded through the thyroid fossa and emerged from the opposite side of the neck wound, securely anchored in place. The wound was subsequently closed in layers, ensuring precise and meticulous closure techniques.

#### ETA

**Fig. 1:**
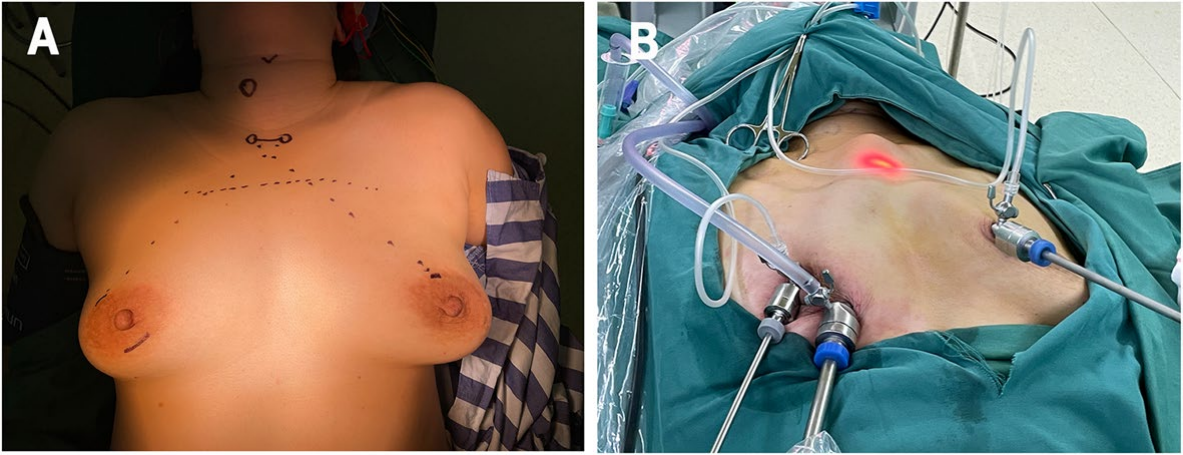
Operative view of ETA. **A** Incision design and surface marking for surgical procedure of ETA. **B** External view after positioning the trocars during ETA. ETA, Endoscopic thyroidectomy via areola approach

Following the induction of general anesthesia, the patient was positioned supine, with their shoulders slightly elevated and legs apart. To initiate the minimally invasive procedure, two small incisions measuring 0.5 cm each were created at the 12 o'clock position on both sides of the areola. These incisions served as access points for 0.5-cm trocars. In addition, a 1-cm incision was thoughtfully prepared at the medial aspect of the right circumareolar area to accommodate a 1.0-cm trocar (Fig. 1A). For the purpose of creating an optimal surgical environment, a solution consisting of 1 mg of epinephrine in 500 ml of normal saline was prepared. Subsequently, 70 ml of the aforementioned solution were extracted and blended with 2 sticks of ropivacaine for injection. This resulting “tumescent solution” was then carefully injected into the subcutaneous tissue of the breast, anterior chest, and the subplatysmal space in the cervical area. A specialized flap dissection stick was employed to delicately separate the deep fascia situated below the suprasternal notch through the previously made incision. Subsequently, a 1.0-cm endoscope was inserted through the 1.0-cm trocar. To create an appropriate working space and facilitate the procedure, carbon dioxide (CO_2_) gas was gently insufflated at a pressure of 6 mmHg with a high flow rate. The subcutaneous loose connective tissue, establishment of the initial working space were completed in order, as depicted in Fig. 1B. Subsequent phases of the procedure closely mirrored those of COT.

#### GTET

**Fig. 2:**
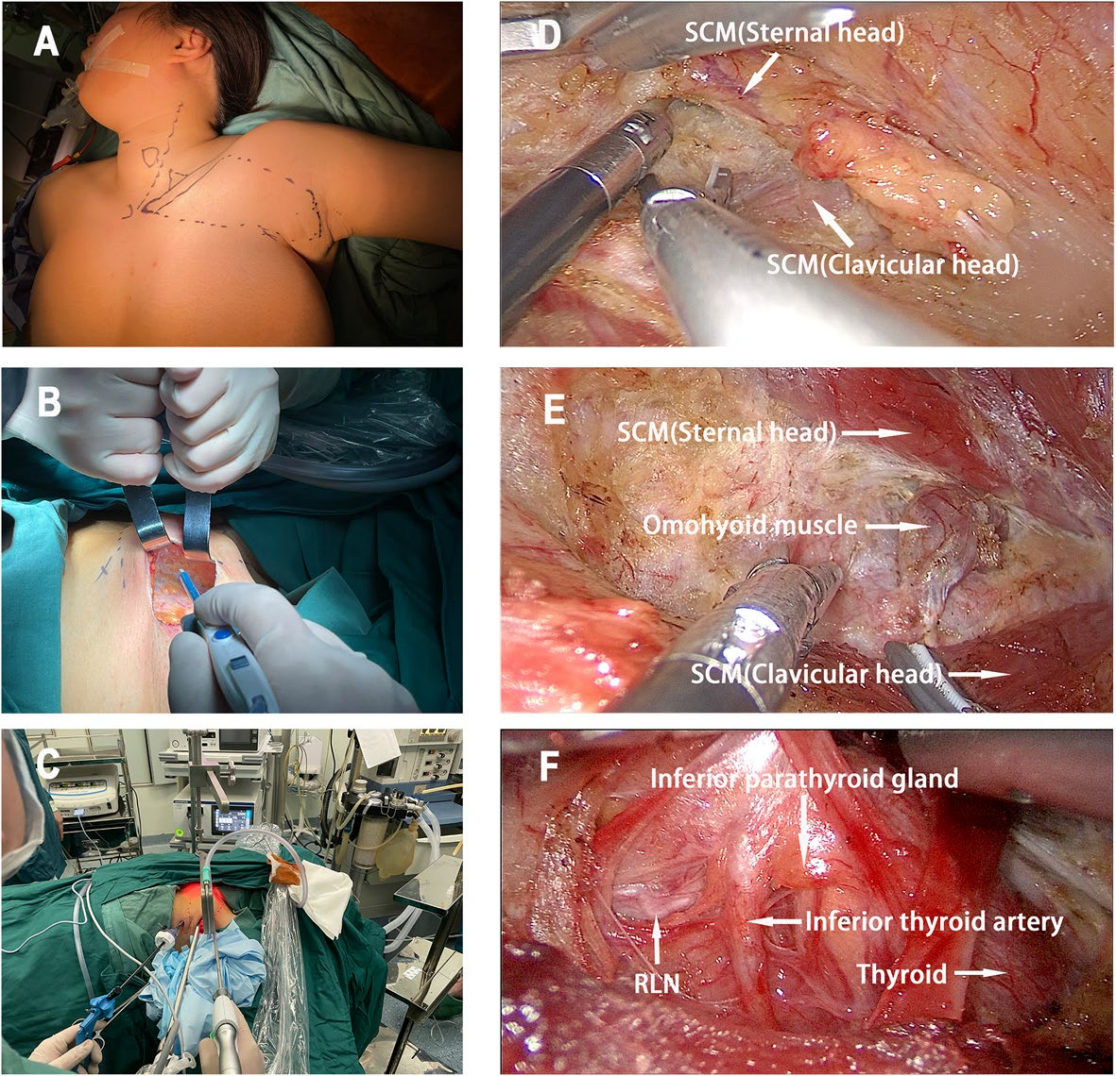
Operative view of GTET. **A** Incision design and surface marking for surgical procedure of GTET. **B** Separation of skin flap under direct vision. **C** External view after positioning the trocars during GTET. **D** Identification and separation of the sternal and clavicular heads of SCM. **E** Exposure of the omohyoid muscle between two heads of SCM. **F** The inferior parathyroid gland, RLN and inferior thyroid artery were identified and exposed during thyroidectomy. GTET, Gasless transaxillary endoscopic thyroidectomy. SCM, Sternocleidomastoid muscle. RLN, Recurrent laryngeal nerve

Upon the administration of general anesthesia, the patient was positioned in the supine orientation, with the affected-side arm elevated and securely immobilized to reveal the axillary region. We employed a modified surgical technique, as introduced by Zhou et al [11]. An incision measuring 4–6 cm in length was thoughtfully executed within the axillary region, ensuring it did not extend beyond the anterior axillary line. Below the initial incision, another incision, measuring 0.5 cm, was skillfully created to accommodate a trocar, as depicted in Fig. 2A. A subcutaneous skin flap was then developed over the pectoralis major muscle, extending to the upper border of the clavicle, as shown in Fig. 2B. Subsequent dissection efforts were aimed at identifying both the sternal and clavicular heads of the SCM, depicted in Fig. 2D. The dissection continued through the natural gap between these two heads of the SCM, with the omohyoid muscle being exposed under retraction, aided by an endoscopic device, as illustrated in Fig. 2E. Following this step, the deep aspect of the strap muscle was separated to expose the thyroid gland. The retractor was then employed to elevate the strap muscle, completing the creation of the surgical cavity, as shown in Fig. 2C. Subsequently, the thyroid lobectomy on the affected side and central neck dissection were executed in a manner akin to COT, as depicted in Fig. 2F. To ensure postoperative drainage, a drainage tube was carefully placed through the axillary incision following adequate hemostasis. The layers of the axillary incision were then meticulously sutured.

### Statistics analysis

CUSUM analysis serves as a valuable quantitative tool for assessing the learning curve [12]. Specifically, it allows for the quantification of cumulative differences between observed data and a predefined target value. In our study, we organized the 72 patients in GTET group chronologically based on their operation dates, ranging from the earliest to the most recent date of surgery. The CUSUM for operation time (CUSUM(OT)) was calculated as follows: CUSUM(OT) = ∑ni (OTi—OTmean), where OTi represented the individual operation time, and OTmean denoted the mean operation time across all cases [13]. Subsequently, we constructed a learning curve using the number of surgical cases on the x-axis and the CUSUM value on the y-axis. We employed Graphpad to fit the CUSUM learning curve. In the CUSUM graph, the point where the slope transitions from positive to negative indicates the point at which the learning curve is successfully surmounted. This vertex of the CUSUM learning curve serves as the cutoff value. Patients were then categorized into two groups based on this cutoff value: group A (≤ cutoff value), representing the learning phase, and group B (> cutoff value), representing the mastery phase. The value on the x-axis corresponding to the vertex of the curve signifies the minimum number of surgeries required to traverse the learning curve.

Chi-square analysis was employed to investigate associations between categorical variables, while nonparametric tests were utilized to explore correlations between continuous variables. Categorical variables were described using the number of cases(percentage), while continuous variables were described using means ± standard deviations and median(quartiles).

For ethical considerations, all patients provided informed consent, which comprehensively detailed the surgical methods and potential complications, including the possibility of conversion to the conventional open approach.

## Results

Table 1 summarizes the clinical and pathological characteristics of the patient cohorts. Both the GTET and ETA groups exhibited a similar proportion of female patients (76% vs. 84%, *P* = 0.218), which was higher than that in the COT group (57%, *P* < 0.001). Furthermore, the mean ages of the two endoscopic groups were notably younger than that of the COT group (*P* < 0.001). No significant disparities were observed among the three groups concerning body mass index (BMI) (*P* = 0.233), duration of postoperative hospital stay (*P* = 0.138), mean duration for surgical drain removal (*P* = 0.262), maximal tumor size (*P* = 0.524), tumor multiplicity (*P* = 0.302), and the incidence of Hashimoto's thyroiditis (*P* = 0.824). Nevertheless, the mean operation time was significantly shorter in the COT group compared to both endoscopic surgery groups (*P* < 0.001). Notably, within the endoscopic surgery groups, the GTET procedure demonstrated a notably reduced operation time in comparison to ETA (125.00 ± 38.00 min vs. 142.10 ± 20.57 min, *P* < 0.001). Furthermore, there were no statistically significant differences among the three groups in terms of the number of retrieved central lymph nodes (*P* = 0.603) and the number of metastatic central lymph nodes (*P* = 0.111).

**Table 1:** Clinicopathologic characteristics of the three groups

Variables	GTET(1)(*n* = 72)	ETA(2)(*n* = 105)	COT(3)(*n* = 394)	*p*-value	(1)versus(2)	(1)versus(3)	(2)versus(3)
Gender							
Female, n(%)	55(76%)	88(84%)	223(57%)	< 0.001	0.218	0.002	< 0.001
Male, n(%)	17(24%)	17(16%)	171(43%)				
Age(years)	36.94 ± 7.25,36(31.25–41)	38.45 ± 8.62,37(31–45)	48.34 ± 12.16,48(38.75–57)	< 0.001	0.33	< 0.001	< 0.001
BMI	23.35 ± 2.29,23.47(21.26–24.63)	23.53 ± 3.71,22.76(20.86–25.38)	23.94 ± 3.94,23.59(21.27–26.54)	0.233	0.61	0.24	0.16
Postoperative hospital stay(days)	3.86 ± 0.88,4(3–4)	4.06 ± 0.63,4(4–4)	3.95 ± 0.67,4(4–4)	0.138	0.091	0.36	0.096
﻿Mean duration for removal of the surgical drain(days)	2.75 ± 0.93,3(2–3)	2.59 ± 0.70,2(2–3)	2.70 ± 0.68,3(2–3)	0.262	0.37	0.8	0.098
Operation time(min)	125.00 ± 38.00,120(95–145)	142.10 ± 20.57,145(125–155)	76.46 ± 22.72,70(60–90)	< 0.001	< 0.001	< 0.001	< 0.001
﻿Maximal tumor size(cm)	0.861 ± 0.52,0.8(0.5–1.0)	0.995 ± 0.65,0.8(0.5–1.3)	0.996 ± 0.66,0.8(0.5–1.3)	0.524	0.26	0.32	0.76
Multiplicity							
No	68	92	358	0.302	0.13	0.318	0.321
Yes	4	13	36				
Hashimoto’s thyroiditis, n (%)							
No	60(83%)	85(81%)	329(84%)	0.824	0.686	0.972	0.537
Yes	12(17%)	20(19%)	65(16%)				
Central lymph node							
No. retrieved lymph node	6.9 ± 2.22,7(5–8.75)	6.56 ± 2.47,6(5–8)	6.9 ± 3.85,6(4–10)	0.603	0.32	0.35	0.93
No. metastatic lymph node	0.96 ± 1.12,0.5(0–2)	1.09 ± 1.64,0(0–2)	1.06 ± 1.99,0(0–1)	0.111	0.72	0.087	0.13

The incidence of complications, including parathyroid damage (*P* = 0.462), temporary hoarseness (*P* = 0.494), postoperative hematoma (*P* = 0.955), and infection (*P* = 0.736), did not display significant variations among the three groups. Over a 6-month follow-up period, no instances of tumor recurrence were detected through neck ultrasonography in any of the three groups (Table 2).

**Table 2:** Postoperative complications of the three groups

Variables	GTET(1)(*n* = 72)	ETA(2)(*n* = 105)	COT(3)(*n* = 394)	*p*-value	(1)versus(2)	(1)versus(3)	(2)versus(3)
∆PTH (pg/ml)	17.06 ± 12.17,14.6(9–22.7)	18.88 ± 13.90,16.3(7–26.45)	16.77 ± 12.24,14.1(7.85–22.53)	0.462	0.5	0.72	0.22
﻿Temporary hoarseness	2 (2.8%)	2 (1.9%)	4 (1.0%)	0.494	1	0.234	0.611
Permanent hoarseness	0	0	0	NA			
Postoperative hematoma	1 (1.4%)	2 (1.9%)	6 (1.5%)	0.955	1	1	0.677
Infection	2 (2.8%)	5 (4.8%)	13 (3.3%)	0.736	0.702	1	0.554
Recurrence(6 months)	0	0	0	NA			

Table 3 presents the results of patients' postoperative pain assessments conducted 3 months after surgery. Among patients who underwent COT, there were 230 reported complaints of neck hypesthesia or paresthesia, which significantly exceeded the occurrences in the GTET and ETA groups. In the endoscopic surgery group, 41 patients in the ETA group reported hypesthesia or paresthesia in the anterior chest, which was notably higher than that in the GTET group (*P* < 0.001). While none of the patients in the COT group reported chest discomfort. Additionally, swallowing discomfort was experienced by only four patients in the GTET group, compared to 22 in the ETA group and 117 in the COT group. The data indicate that GTET significantly reduced swallowing discomfort compared to the other two groups. Furthermore, cosmetic outcomes were superior in the GTET and ETA groups in comparison to the COT group, with statistically significant differences observed at the 3-month postoperative evaluation (*P* < 0.001, *P* < 0.001).

**Table 3:** Patient complaints 3 months after surgery of the three group

Variables	GTET(1)(*n* = 72)	ETA(2)(*n* = 105)	COT(3)(*n* = 394)	*p*-value	(1)versus(2)	(1)versus(3)	(2)versus(3)
Hypesthesia or paresthesia in the neck							
No	65	98	164	< 0.001	0.459	< 0.001	< 0.001
Yes	7	7	230				
Hypesthesia or paresthesia in the anterior chest							
No	63	64	394	< 0.001	< 0.001	< 0.001	< 0.001
Yes	9	41	0				
Discomfort in swallowing							
No	68	83	277	< 0.001	0.004	< 0.001	0.076
Yes	4	22	117				
﻿Cosmetic satisfaction	1.08 ± 0.28,1(1–1)	1.21 ± 0.51,1(1–1)	1.63 ± 0.94,1(1–2)	< 0.001	0.11	< 0.001	< 0.001

**Fig. 3:**
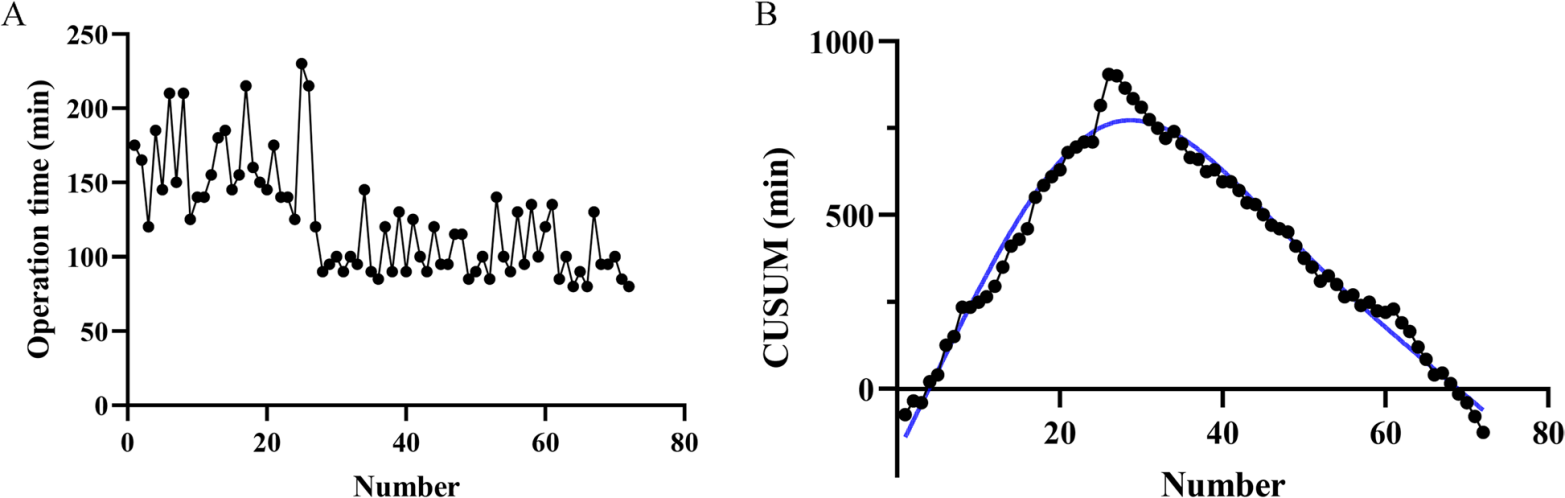
**A** Operation time plotted in chronological order of patients underwent GTET. **B** CUSUM test for operation time of GTET. GTET, Gasless transaxillary endoscopic thyroidectomy. CUSUM, Cumulative summation

**Fig. 4:**
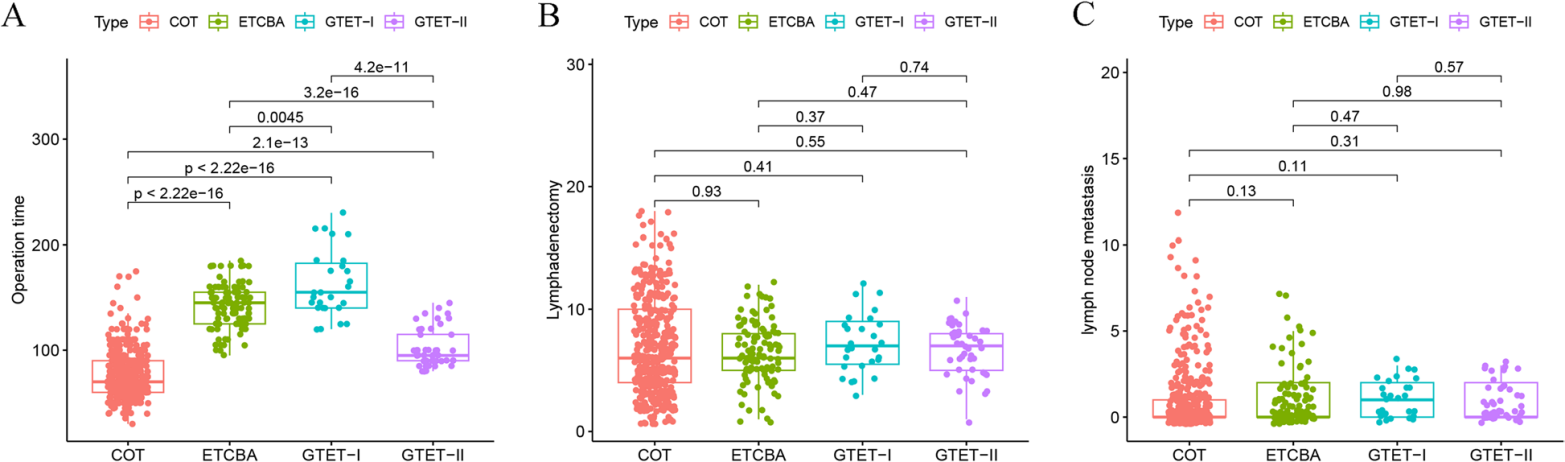
GTET was divided into GTET-I (the learning period) and GTET-II (the mastery period) according to the cutoff value. Surgical outcomes were further compared among the COT, ETA, GTET-I and GTET-II. **A** Comparison of operation time among the four groups. **B** Comparison of the number of retrieved central lymph nodes among the four groups. **C** Comparison of the number of metastatic central lymph nodes among the four groups. GTET, Gasless transaxillary endoscopic thyroidectomy. COT, Conventional open thyroidectomy. ETA, Endoscopic thyroidectomy via the areola approach

The mean operation time for GTET was 125 min. A visual representation of the change in operation time over the course of surgeries indicated a consistent downward trend with increasing case volume (Fig. 3A). According to the CUSUM method, the learning curve for GTET reached its peak at 27 cases (Fig. 3B). Subsequently, patients were categorized into two groups based on the inflection point: GTET-I, representing the learning phase (cases 1–27), and GETE-II, representing the mastery phase (cases 27–72). A comparison of operation time, the number of retrieved central lymph nodes, and the number of metastatic central lymph nodes among the COT, ETA, GTET-I, and GTET-II groups is illustrated in Fig. 4. There were no significant differences observed in the number of retrieved central lymph nodes and the number of metastatic central lymph nodes among the four groups (Fig. 4B-C). In terms of operation time, although the GTET-II group still required more time compared to the COT group, it exhibited a significant reduction compared to the ETA group and the GTET-I group (Fig. 4A).

## Discussion

In the pursuit of enhanced cosmetic outcomes, the field of endoscopic thyroid surgery has experienced rapid growth. And the breast approach has gained substantial popularity, particularly in China [14, 15]. Our institution boasts extensive experience in both COT and ETA and introduced GTET as a novel approach three years ago. To facilitate more equitable comparisons among these three surgical approaches, we limited our study to cases of unilateral thyroid cancer.

It is noteworthy that the cohorts under scrutiny in our retrospective study exhibited significant differences in terms of gender and age. This type of bias has been observed in numerous previous studies [16–18]. The divergence primarily stems from the heightened emphasis placed on aesthetic outcomes among younger individuals, particularly females. The feasibility and reliability of endoscopic approaches in retrieving lymph nodes from the central neck region have been debated [17, 19–21]. The limitations in terms of field of vision and surgical maneuverability in endoscopic groups are attributed to potential visual obstructions and instrument interference caused by the clavicles and sternum barbell. No differences were observed in the number of retrieved central lymph nodes in our study, aligning with the findings of the majority of studies [22–26]. Drawing from our institution's experience, endoscopic dissection of central lymph nodes has its own advantages. On the one hand, a standard 30° endoscope can offer a detailed endoscopic view of the central compartment [27]. On the other hand, during ETA, after severing the thyroid gland isthmus, the central lymph nodes can be dissected and lifted upwards, connecting them with the free thyroid gland. This approach facilitates the complete exposure and visualization of central lymph nodes in conjunction with surrounding fat tissues. In GTET, the lateral perspective makes it easier to remove the central lymph nodes in comparison. Furthermore, GTET excels in exposing the carotid artery, which translates to clear visualization of the lateral junction of central lymph nodes, ensuring comprehensive dissection.

The assessment of technique safety was conducted through various means. The ΔPTH, indicating parathyroid and its blood supply damage, exhibited no variation among the three groups. Additionally, there were no differences in the incidence of RLN palsy, postoperative hematoma, or infection. While there may be slightly higher incidences of RLN and parathyroid gland damage in the endoscopic group due to thermal damage from the ultrasonic scalpel [28], the benefit of endoscopic magnification allows surgeons with adequate training to dissect RLN and parathyroid glands safely, particularly during GTET where lateral visualization is similar to open surgery. Based on these results, we assert that ETA and GTET are safe procedures when performed by experienced surgeons. This conclusion aligns with previous studies [17, 19, 24, 29, 30].

The reform in surgical approaches has improved postoperative patient comfort. Our study found higher incidences of postoperative neck hypesthesia or paresthesia in open surgery compared to endoscopic groups, likely due to the cervical skin incision itself and the adhesions that may develop between the scar and the subcutaneous neck tissue. The psychological impact of a cervical scar could also affect the subjective assessment of postoperative sensation. Otherwise, GTET procedures showed reduced hypesthesia or paresthesia in the anterior chest compared to ETA, possibly due to the transaxillary approach avoiding extensive flap separation. Japanese scholars have reported that keloids tend to form in anatomical areas subjected to mechanical forces from body movement, with the chest being the most common site (42.7%) among 1034 anatomical regions of the human body [31]. This may explain the subjective sensation of chest discomfort following ETA. The transaxillary approach also minimizes discomfort during swallowing, since the entire process takes place within the natural gap of the neck without disrupting the cervical white line. This prevents adhesion between the strap muscle and the subplatysmal muscle flap that may occur during ETA and COT procedures [32]. Furthermore, both endoscopic groups exhibited superior cosmetic satisfaction compared to the conventional group at the 3-month postoperative evaluation, with no significant differences observed between the ETA and GTET groups. Remote-access thyroidectomy resulted in excellent postoperative cosmetic outcomes, supported by previous research [9, 33–35].

ET is technically demanding and typically takes more time than conventional open thyroidectomy [22, 24, 30, 33], as confirmed by our study. Given that we introduced GTET within the past three years while having performed over 500 ETA surgeries spanning more than eight years, we focused on analyzing the learning curve of GTET and compared it further with ETA. Using the CUSUM method, we found that a surgeon requires 27 cases to become proficient with GTET. The inflection point of GTET in our study occurs earlier than other reported studies [36, 37]. This discrepancy may be attributed to the surgeon's extensive experience in both open and other ET approaches, such as ETA.

GTET was divided into two phases: the learning period and the mastery period, delineated by the peak point on the learning curve. This stratification allowed for a rigorous comparison with ETA and COT, minimizing potential deviations resulting from the initial unfamiliarity with the new technique. During the mature phase of GTET, operation times stabilized and efficiency improved compared to the learning period, although it still took longer than COT. However, the mature GTET operation time was shorter than that of ETA, attributed to the elimination of CO2 inflation and lens cleaning requirements. The number of lymph node dissections in GTET remained consistent across different phases and was comparable to the other surgical approaches, reaffirming the surgical efficacy of GTET, regardless of the learning phase.

Several limitations should be considered in the context of this study. Firstly, it was not a randomized study, and the treatment strategy was influenced by patient preference. Secondly, disparities in mean age and gender ratio between the ET and COT groups potentially impact patient subjective assessments of cosmetic outcomes and overall satisfaction. Thirdly, the sample size in this study was relatively modest, and the follow-up duration for assessing recurrence rates was relatively short. Therefore, it is imperative that future research endeavors encompass larger, randomized, and long-term studies to provide a more comprehensive understanding of these surgical approaches.

## Conclusion

In summation, our findings underscore the safety and feasibility of GTET and ETA in the treatment of patients with PTC. Both approaches offer compelling cosmetic advantages. However, GTET stands out for its reduced impact on neck and anterior chest tissue, as well as its potential for greater time efficiency in its mature phase.

## Data Availability

No datasets were generated or analysed during the current study.
